# Design and construction of a novel measurement device for mechanical characterization of hydrogels: A case study

**DOI:** 10.1371/journal.pone.0247727

**Published:** 2021-02-25

**Authors:** Shayan Shahab, Mehran Kasra, Alireza Dolatshahi-Pirouz

**Affiliations:** 1 Tissue Engineering Laboratory, Biomedical Engineering Faculty, Amirkabir University of Technology-Tehran Polytechnic, Tehran, Iran; 2 Department of Health Technology, Institute of Biotherapeutic Engineering and Drug Targeting, Center for Intestinal Absorption and Transport of Biopharmaceuticals, Technical University of Denmark, Kgs Lyngby, Denmark; LAAS-CNRS, FRANCE

## Abstract

Natural biopolymer-based hydrogels especially agarose and collagen gels, considering their biocompatibility with cells and their capacity to mimic biological tissues, have widely been used for in-vitro experiments and tissue engineering applications in recent years; nevertheless their mechanical properties are not always optimal for these purposes. Regarding the importance of the mechanical properties of hydrogels, many mechanical characterization studies have been carried out for such biopolymers. In this work, we have focused on understanding the mechanical role of agarose and collagen concentration on the hydrogel strength and elastic behavior. In this direction, Amirkabir Magnetic Bead Rheometry (AMBR) characterization device equipped with an optimized electromagnet, was designed and constructed for the measurement of hydrogel mechanical properties. The operation of AMBR set-up is based on applying a magnetic field to actuate magnetic beads in contact with the gel surface in order to actuate the gel itself. In simple terms the magnetic beads leads give rise to mechanical shear stress on the gel surface when under magnetic influence and together with the associated bead-gel displacement it is possible to calculate the hydrogel shear modulus. Agarose and Collagen gels with respectively 0.2–0.6 wt % and 0.2–0.5 wt % percent concentrations were prepared for mechanical characterization in terms of their shear modulus. The shear modulus values for the different percent concentrations of the agarose gel were obtained in the range 250–650 Pa, indicating the shear modulus increases by increasing in the agar gel concentration. In addition to this, the values of shear modulus for the collagen gel increase as function of concentration in the range 240–520 Pa in accordance with an approximately linear relationship between collagen concentration and gel strength.

## I. Introduction

Biological tissues are of great importance in mechano-transduction studies because they that provide the mechanical microenvironment for cells. However, their experimental characterization is quite complicated and it is not always possible to work with them. Thus, the provision of human healthcare facilities is pertinent to development of sustainable strategies and multifaceted engineering biomaterials that can address tissue engineering requirements as an emerging endeavor. In the last decades, biopolymer-based hydrogels have become one of the best alternatives to biological tissues in cell culture and for tissue engineering [1, 2].

In brief, hydrogels are biocompatible polymeric biomaterials that due to their water-swollen cross-linked network and soft, rubbery consistency, have a strong resemblance to living soft tissue. Indeed, native-like hydrogels can mimic the natural support structures used by the human body to guide the behavior of cells within tissues [3, 4]. In this direction, tissue engineering applications generally require the use of a biodegradable scaffold serving as a 3D template for cell attachment and subsequent tissue formation and thus, soft hydrogels have widely been used by many investigations as substrate for cell culture in tissue regeneration and mechanical manipulation of different cell types [5–7].

There has been tremendous growth also in the area of hydrogels for drug delivery applications due to their important qualities such as biocompatibility and biodegradability. In this regard, a new kind of arginine-based hybrid hydrogel was developed to perform transdermal drug delivery [8]. The article [9] reviews the recent developments of Cyclodextrin-based supramolecular hydrogels in injectable drug delivery to provide a new platform for the design of novel hydrogel-based biomaterials. Jun Wu et al. [10] reported the development of a biodegradable hybrid hydrogel platform for controlled release of ionic drugs. A newly fabricated Amino-Acid-based cationic hybrid hydrogel, demonstrating a greatly improvement the attachment of human fibroblasts on hydrogel surface, was reported in [11].

One of the most limiting factor associated with hydrogel materials, when they are *in-vitro* used in tissue engineering and drug delivery, is their relatively poor mechanical properties. Indeed, the most challenging issue in employing hydrogels as cell culture substrates, biomimetic materials, and matrices for regeneration of a wide variety of tissues is their biomechanical features that can be tailored in a wide range of soft to hard tissues with suitable stability in physiological milieu for achieving the biopolymer-based substitutes showing closest mechanical characteristics to those of natural tissue [12, 13]. Therefore, numerous experimental methods have previously been employed to characterize the mechanical properties such as tensile strength and elastic modulus of hydrogels through either tensile testing [14] or tapping into the micro-indentation technique [15]. With the advancement of technology, more sophisticated techniques such as Atomic Force Microscopy (AFM) [16], texture analysis [17], optical tweezers [18], and laser tracking microrheology [19] have been used to characterize the mechanical properties of hydrogels.

Hydrogels can be classified as natural and synthetic and the use of Naturally-occurring polymeric gels, such as Agarose and Collagen, in view of their non-toxic nature as well as satisfactory compatibility with various tissues/organs, has opened promising windows in front of tissue engineering and healthcare-related applications [20, 21]. Specifically, Agarose is a natural polysaccharid polymer having unique characteristics such as biocompatibility, thermo-reversible gelatin behavior, and physiochemical features that support its use as a biomaterial for cell growth and controlled drug delivery. Agarose-based scaffolds for targeted tissues can be achieved via hydrogels [22], injectable hydrogels [23], self-healing hydrogels [24], and 3D printed scaffolds [25] which can combine with other materials to form a hybrid platform with high performance like fibrin-agarose [26]. In addition to this, agarose hydrogel is considered as the most commonly utilized brain mimicking material for enhancing the rate of drug delivery to brain tissue in Ultrasonic Field Enhanced Drug Delivery (UED) technique [27, 28]. Since the mechanical properties of the gel are quite sensitive to the concentration of the agar powder in water, previous investigations have studied the mechanical properties of agarose gel at different percent concentrations [29, 30]. In this regard, Luke Varkey [31] studied the diffusivity of dye through agar gel brain phantom with an aim to understand drug diffusion in brain tissue using 0.5 wt % agar gel concentration. Fallenstein et al. [32] have studied the shear modulus of in-vitro samples of human brain and then compared these values with obtained shear modulus of agar gel samples. R. Deepthi et al. [33] also have a rheometric study on agarose gel for measurement of its shear modulus parameter under the applied mechanical vibrations.

Likewise, collagen-based hydrogels have been widely used in tissue engineering application due to their biocompatibility with cells and their capacity to mimic biological tissues. In this direction, collagen gels are gaining widespread popularity as scaffolds for tissue engineering due to the abundance of collagen in natural ECM. The key element in using this type of hydrogel is collagen concentration which in-vitro and in-vivo influences tissue mechanical properties, thereby regulating cellular behavior [34, 35]. Therefore, mechanical characteristics of collagen hydrogels have to be measured to assure their similarity to biological tissues. The variation of the mechanical properties of collagen hydrogels with different collagen concentration was studied in refs [36] and [37]. Dewi Harjanto et al [38] have a research on quantitative analysis of the effect of collagen concentration on 3D matrix remodeling. Clara Valero et al [39] have also analyzed the mechanical implications of varying the collagen-based concentrations and adding a crosslinking agent to collagen hydrogels.

The grand aim of the present study is to characterize the mechanical properties of elastic polymeric hydrogels in a non-destructive fashion. In this research, considering the importance of the mechanical characteristics of hydrogels, the elastic shear modulus of the agarose and collagen hydrogels of various concentrations was studied using a novel characterization device called Amirkabir Magnetic Bead Rheometry (AMBR) has been developed for the measurement of hydrogel mechanical properties. Specifically, an apparatus was developed, that allows the determination of the local gel elasticity, by measuring the response of the placed magnetic bead on the gel surface to externally applied magnetic fields.

### I-A. Statement of significance and novelty

Mechanical properties of hydrogels are important design parameters to be considered for engineering of hydrogels from pharmaceutical and biomedical point of view [40]. Mechanical characteristics of gels have been shown to influence important biological properties such as adhesion and cell spreading [41], migration [42], proliferation [43], and differentiation [44]. The biomechanical feature of gels depend on many factors, mainly the hydrogel composition, preparation method, and environmental conditions. In this direction, elastic modulus of hydrogels specifically hydrogel shear modulus is a critical property for understanding hydrogel mechanical behavior for achieving bioengineered structures with the desired mechanical characteristics. Several techniques such as AFM, texture analysis, rheometry and indentation-based methods have broadly been used for mechanical characterization of hydrogels. In this regard, texture analyzer allowed measurement of gel performance under surface normal forces, and thus has been used to characterize the gel hardness, cohesiveness, and adhesiveness.

Atomic Force Microscopy technique is also used to determine local mechanical properties of hydrogels through measuring the force versus distance at a specific point on the gel surface. When compared to rheometer, AFM is often used for scanning the surface of gel/cell sample in constant frequency rates using cantilever-based tip to measure and localize many different forces such as mechanical properties. However, Rheology-based techniques, will allow user to evaluate gel or cell sample viscoelastic behavior accompanying with time-dependent response. Rheometry set-up is thus utilized for applying a determined range of frequencies to cell-bead sample for mechanical characterization in terms of creep response or complex modulus. Conventional indentation techniques even AFM are also limited for use in sterile environments under cell culture conditions [45–48].

In addition to this, most of these techniques are either intrusive or destructive in nature. In particular, using traditional methods like indentation for measuring the mechanical properties of the gels will be of limited utility for microliter-sized samples that are mounted on a microscope with cells attached to the surface of the gels; however the set-up of this work will allow us to characterize both of cell and hydrogel mechanical properties in terms of complex or shear modulus without such limitation in prepared gel-cell sample dimensions. Such methods are also of limited usefulness when stiffness or elastic modulus varies with position and time within the gel. In this case, the placed magnetic bead on hydrogel surface can be actuated non-intrusively using an external magnetic force.

The significant aim of this work is to characterize the mechanical properties of hydrogels in condition showing the most similarities to cell culture media. Indeed, this work employs a sample preparation protocol that permits measurements in conditions closer to those in-vivo and the set up was thus calibrated in water having the closest viscosity value to cell culture media. This means the set up can be used for the measurement of hydrogel mechanical properties particularly gel shear modulus with an aim in achieving the biopolymer-based substitutes either demonstrate mechanical/ biological characteristic resemble to cell or mimic the cell natural behavior for predicting cell responses.

In several previous researches, the utilized rheometer device just could generate magnetic forces up to 10 nN which was used for mechanical characterization of specific cell types. To overcome this challenge in existing technique to circumvent the limitation associated with the maximum force that can be generated by the Rheometer device, the technique utilizes a new electromagnet set-up equipped to an optimized core tip which not only do generate magnetic forces up to 50 μN for actuation of gel surface, but also direct the generated magnetic force in the vicinity of magnetic particle. In this case, we used CST simulation software for calculation of the generated magnetic field in the vicinity of magnetic particle, allowing us to determine the optimized distances between core tip and magnetic bead for generation of effective magnetic forces. The precise alignment of magnetic particle and electromagnet will generate the focused magnetic field toward magnetic bead for maximizing the generated magnetic force, leading to the enhancement of Rheometer set-up efficiency.

Eventually, as industrial point of view, the set-up of this research is a very low-cost device as compared with similar counterparts, made up of low-priced components. In addition to this, as engineering viewpoint, the portable and weightless AMBR set-up, having simple circuit design beside no need to operational preparation process, is suggested as appropriate and practical mechanical characterization device for educational purposes as compared with the sophisticated instruments.

The aim of the present work in particular is determining the agar gel concentration that give rise to mechanical properties closest to that of brain tissue. The obtained shear modulus values are then compared with those of human brain tissue reported in the literature. We have also analyzed the mechanical implications of varying the collagen-based concentration in order to specify the desired collagen gel strength for employing in tissue engineering especially in cell culture systems.

## II. Design and simulation

In previous studies, due to using simple cylindrical coils, low-intensity magnetic fields were generated in the vicinity of the magnetic particle. Therefore, the employment of two or more magnetic coils were needed for providing the required magnetic field values. In this research, we specifically aimed at optimizing the coil operation by using a new electromagnet equipped to a conical core instead of standard cylindrical coils typically used in the field. Specifically, a magnetic coil with an internal conical core tip was used in order to focus the generated magnetic field toward the magnetic particle. In brief, the electromagnet consists of an aluminum frame with the length and diameter of respectively 45 mm and 20 mm into which a soft-iron core of 14 mm diameter was fixed.

**Fig 1:**
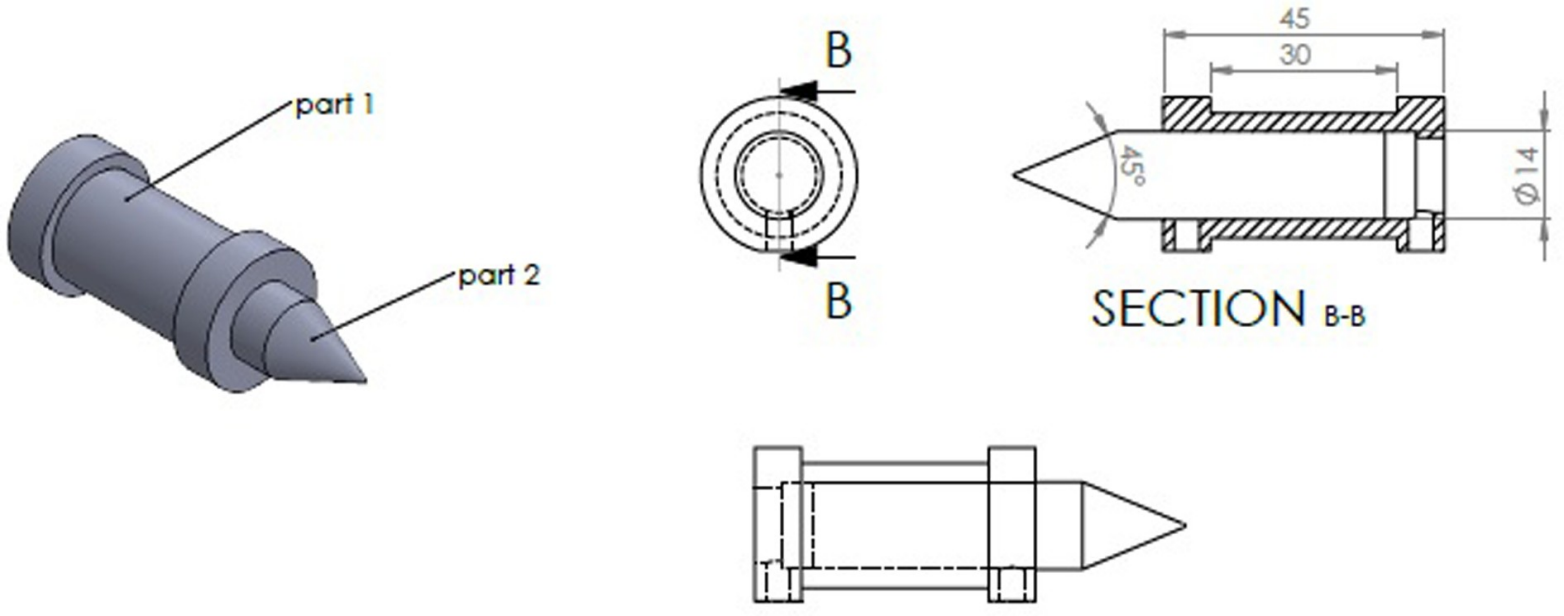
The 3D schematic of the designed electromagnet by SOLIDWORKS software; part 1: Cylindrical coil, part 2: Magnetic field focusing core tip (pole piece).

The magnetic core tip directed the generated magnetic field toward the magnetic bead in a 45° focusing angle. Considering the coil length, the 50 turns of 0.6 mm copper wire were wrapped around the coil cylindrical frame for the generation of the appropriate magnetic field intensity. The 3D image of the electromagnet, designed by the SOLIDWORKS software, was shown in Fig 1.

The magnetic field simulation by using CST software was then employed for the investigation of the pole piece effect and also for the calculation of the magnetic field intensity in specific distances to the core tip. This allowed us to determine the distances between core tip and magnetic bead for generation of effective magnetic forces. Simulation results then help us to select the optimized magnetic bead size regarding hydrogel sample type and dimensions.

**Fig 2:**
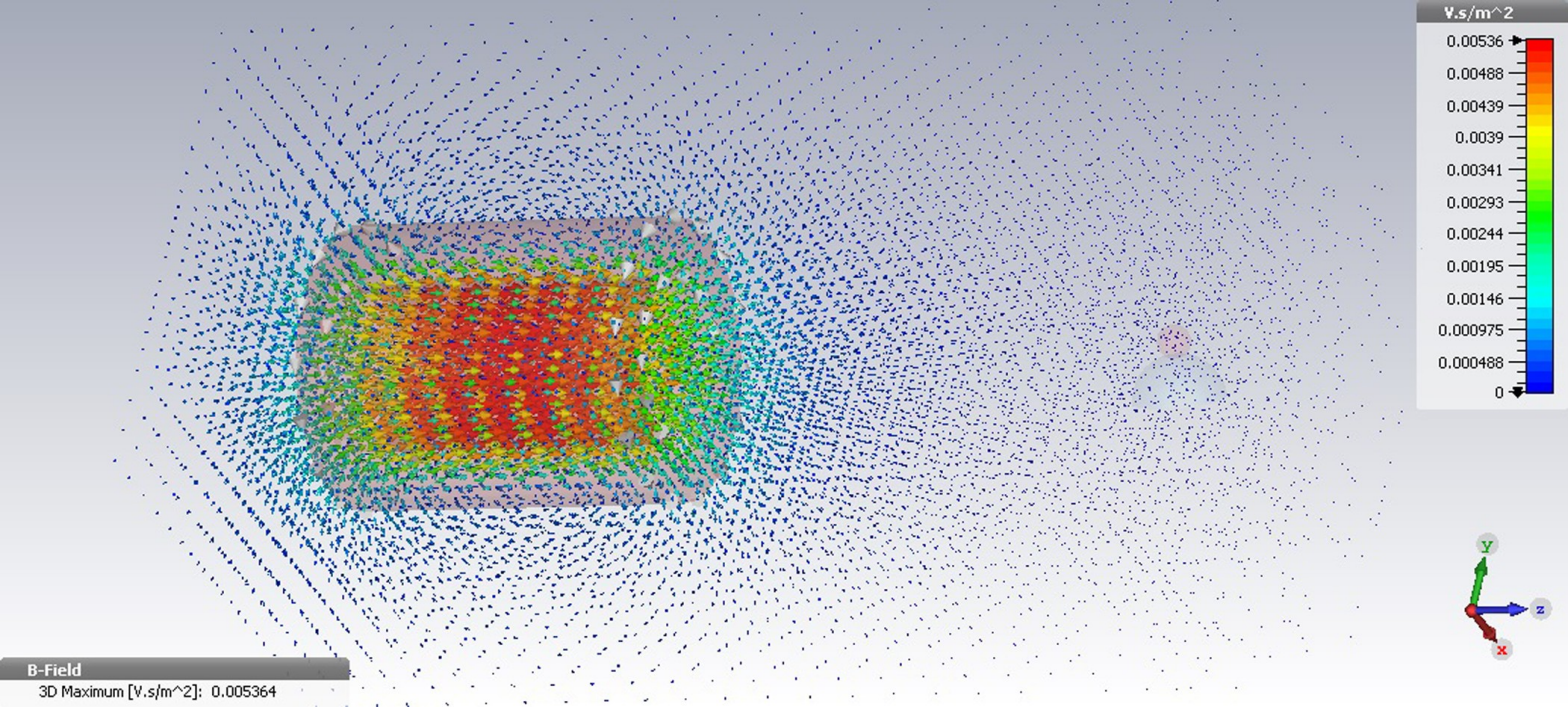
The 3D image of magnetic field simulation for the cylindrical coil under the applied current 3000 mA using CST.

For this, the given values of electric current were provided to the designed electromagnet with the same dimension and turn number use in the CST simulation. The magnetic field intensity was then calculated in the vicinity of each two coils. First, the magnetic field simulation for common cylindrical coil consisting of the 50 turns of copper wire was carried out using 2000 mA and 3000 mA current values. In the simulation of the applied 2000 mA and 3000 mA currents, the maximum value of the generated magnetic field by the cylindrical coil was respectively obtained at 8 G and 12 G. Notably, in this scenario the magnetic field intensity significantly decreases by increasing in distance from the coil pole tip. Fig 2 shows the 3D image of the cylindrical coil simulation with the generated magnetic field lines under the provided 3000 mA current.

**Fig 3:**
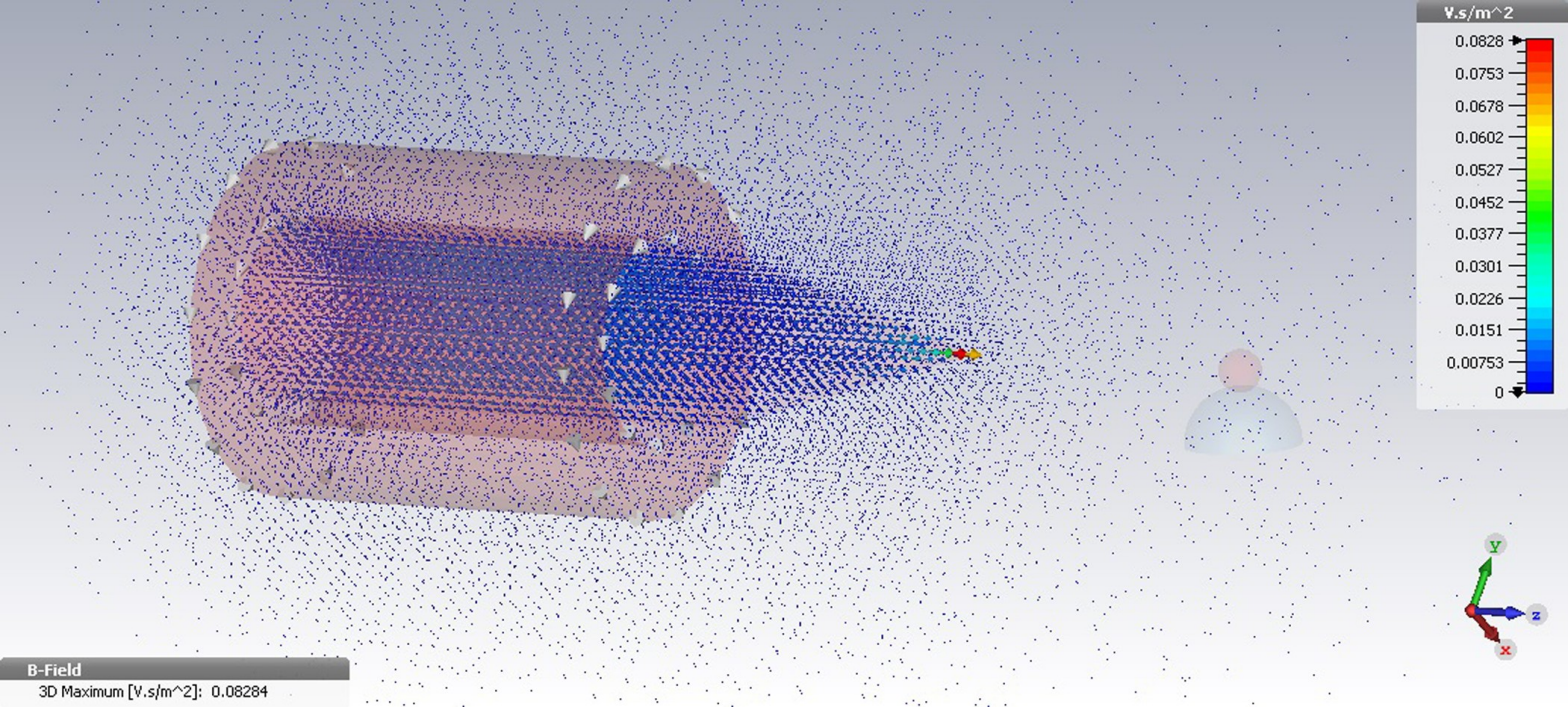
The 3D image of magnetic field simulation for the designed electromagnet equipped to a conical core tip under the applied current 3000 mA using CST.

The magnetic field simulation was then performed in CST software for the designed electromagnet, which was equipped to a conical-shaped core tip in the same current values. The magnetic field intensity in the vicinity of the magnetic particle, under the provided 2000 mA current, was obtained at 35 G indicating over four times increase in the magnetic field intensity than the former case. In addition to this, it was observed in the Fig 3 that the magnetic field intensity in distances of 1–3 mm to the magnetic particle under the provided 3000 mA current increases up to 60 G–an approximately five times in the magnetic field strength as compared with the simple cylindrical coil.

Therefore, considering the simulation results, the soft-iron core tip focus the generated magnetic field toward the magnetic particle leading to the generation of high intensity magnetic forces in the specific distances to the pole piece. In addition to this, the simulation results also indicate that the precise alignment of core tip with the magnetic bead lead to generation of the maximum value of focused magnetic force on particle. Thanks to this special design, the appropriate values of the magnetic field intensity can be obtained in the vicinity of magnetic bead in the lower applied currents, increasing the operational efficiency of the single coil magnetic systems.

## III. Materials and methods

The microscope-based magnetic bead rheometer with its main components are discussed in detail in following. The force calibration of the rheometer set-up in order to determine the optimized operational condition of the device is also mentioned in this section. The utilized biopolymer-based hydrogels beside their preparation method and the employed mechanical characterization technique for gel mechanical properties measurement are eventually explained in the rest of this section.

### III-A. Amirkabir Magnetic Bead Rheometry (AMBR) set-up

**Fig 4:**
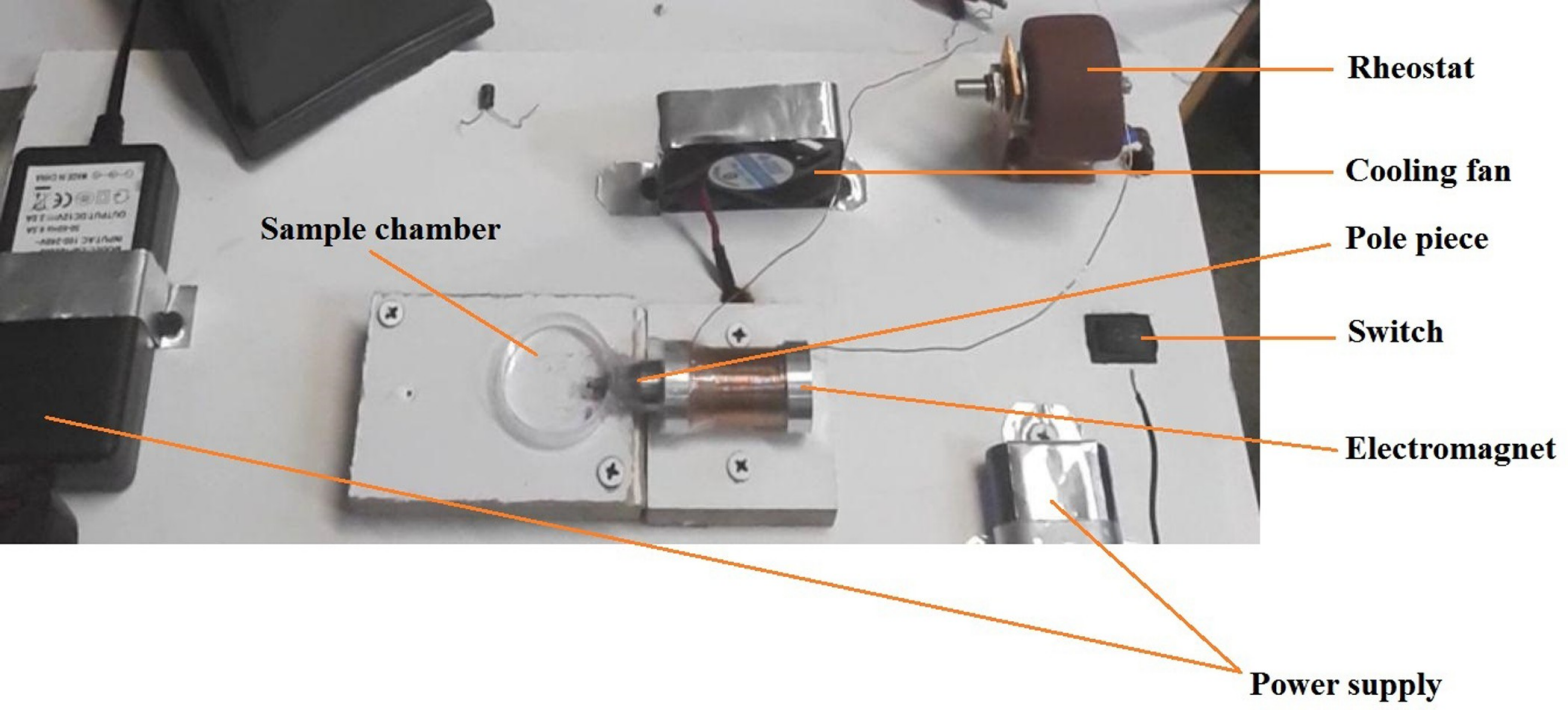
The image of the constructed AMBR set-up with the main components.

In this research, Lithium chargeable batteries of I = 2.2 Ah, V = 3.8 V that were connected in a parallel configuration, were used as current supply for the generation of electric currents up to 5 A. Also, a variable resistor of 5R6, with the total resistance of 5.6 Ω, was utilized for controlling the provided electric current to the electromagnet. The designed electromagnet with considering the simulation characteristics was constructed for the generation of magnetic field and was then fixed into holder panel. The electromagnet was designed vertically variable and therefore, core tip can be accurately aligned with magnetic particle in order to maximize the generated magnetic force in the vicinity of gel sample. In addition, a cooling system which consists of a FAN of 6*61.5 cm, electrically connected to a power supply of I = 2 A, V = 12 V, was employed for preventing the coil internal resistance increase. In this research, a magnetic spherical particle of 1000 μm diameter which was placed in a polystyrene holder of 3 cm diameter, was utilized for the experiments. The provided components were fixed onto the holder panel regarding the appropriate configuration and were then electrically connected to each other for the set-up operation. The magnetic core tip was penetrated to the sample holder by which the magnetic bead was directly exposed to the generated magnetic field; thus, the intensity of the magnetic force acting on the magnetic particle significantly increases. Also, considering the operational efficiency of the set-up based on the results of simulations, the magnetic particle was placed in the distances of 1–3 mm to the electromagnet core tip in order to maximize the value of the applied magnetic field to the particle. Fig 4 shows the image of the constructed AMBR device with the main components.

### III-B. Force calibration of the set-up

To calibrate the distance dependence of the force acting on the magnetic particle, while we have precise alignment of pole piece with magnetic particle, the bead velocity near the core tip was determined in liquids of known viscosity at different coil currents. The bead velocity was computed from the measured displacement–time graphs by numerical differentiation. The velocity curves were converted into force curves using Eq 1, known as Stokes law.

F=3πμmDV (1)

In which μ, D, V, and F are respectively the magnetic bead diameter, liquid viscosity, bead velocity, and the magnetic force which act on the particle.

In this research, force calibration for the magnetic particle of 1000 μm was performed in different electric currents ranging from 2500 mA to 4000 mA in distances of 1–3 mm to magnetic core tip regarding simulation results. We used water with the viscosity of approximately 1–1.2 Kg/m.s, the most similar viscosity to the cell culture media containing 10% FBS, as calibrating liquid. To determine the magnetic bead velocity in liquid, the particle displacement toward electromagnet was measured versus displacement time. Also, the value of the applied current to electromagnet, leading to the generation of magnetic field, was experimentally measured by using the provided multimeter. Therefore, the magnetic force which act on the magnetic bead due to the applied magnetic field, can be calculated from the Stokes law. We then used A1322 Hall Effect Sensor for measurement of the generated magnetic field by electromagnet with an aim to minimize the probable errors of simulations [49]. This consequently helped us in experiments to correct the magnetic bead distance to pole-piece regarding error indices. Indeed, the obtained magnetic field values of CST simulation was checked by Hall-effect sensor and thus, the accurate magnetic field values generated in experimental condition was determined. This enable us to ensure that the required magnetic field was practically generated and hence, this would lead to the generation of required mechanical forces on the gel surface for calculation of shear modulus parameter.

**Fig 5:**
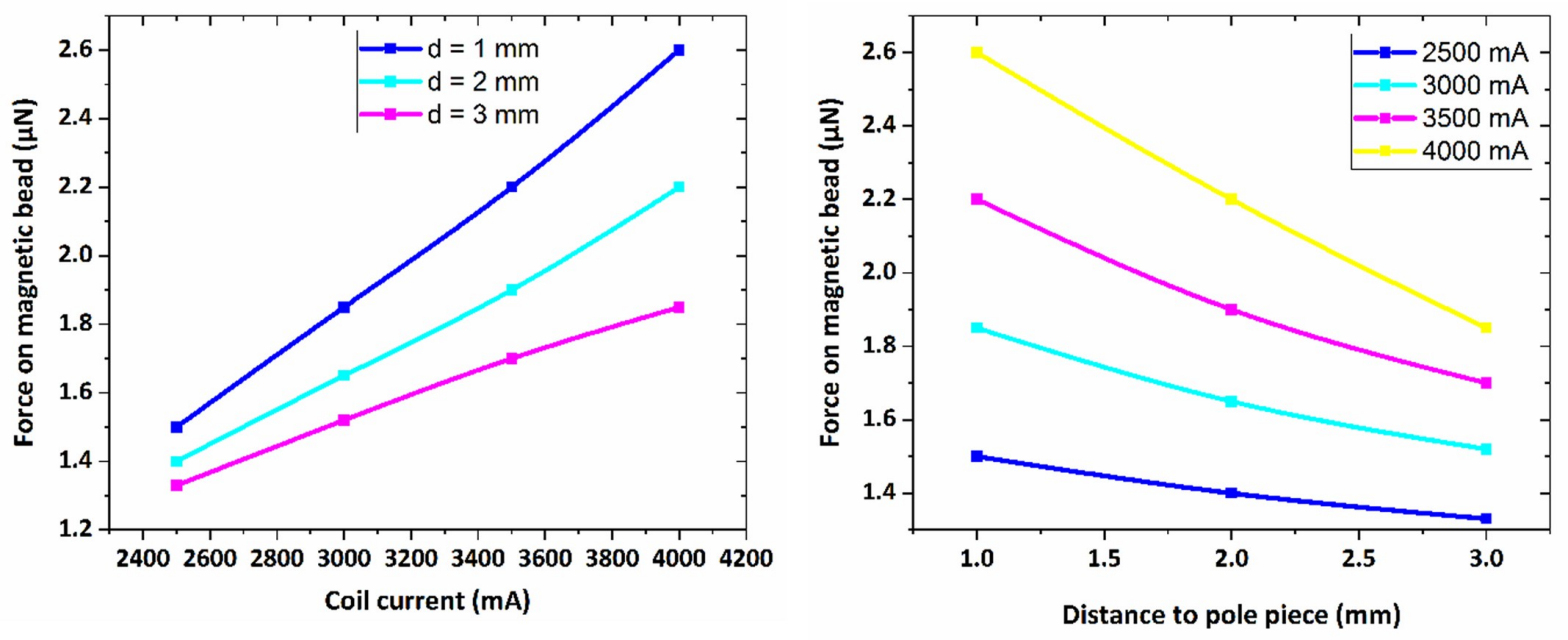
The graph of magnetic force versus coil electric current for distances of 1–3 mm from pole piece for the bead of 1000 μm.

In Fig 5, the magnetic force is plotted versus the coil current for different distances from the core tip. As seen in figure, there is a linear dependence between the magnetic force and the applied current to the electromagnet indicating the utilized bead is fully magnetized in the presence of magnetic field in water. Indeed, at a specific distance from the electromagnet core tip, the applied force on the bead of 1000 μm diameter increases by increasing in the value of the provided current to the electromagnet.

### III-C. Preparation of hydrogels

In this study, two types of agarose and collagen hydrogel of different gel concentrations were used for their shear modulus measurement. Both agarose and collagen were first provided in commercially available powder and then, according to the standard methods explained in the ensuing lines, were prepared in the gel form for experimental testing.

The Agarose gel with percentage concentrations (weight / volume, W/V) of 0.2 wt %, 0.3 wt %, 0.4 wt %, 0.5 wt %, and 0.6 wt % were prepared by dissolving the appropriate amount of agar gel powder, weighed accurately using an electric balance, into the distilled deionized water. The solution was sealed and heated to 90–95°c for about 15 minutes and was magnetically stirred to be homogenous. The prepared solution, poured into the provided micro tube, was then allowed to cool for 5 to 6 hrs to form a gel. The small hemispherical gel samples of about 3–4 mm diameter were cut and used for the measurements.

Acid solubilized type I Rat Tail Collagen (RTC) was aliquoted under sterile conditions and stored at 4°c until required. A neutralization solution was prepared by using the appropriate volumes of 10× Dulbecco’s Modified Eagle’s Medium (DMEM) (Gibco), 0.925 M NaOH (Fluka), sterile water, Fetal Bovine Serum (FBS) (Gibco) and 1× DMEM (Gibco) to mix with the required volume of RTC. Then, the pH 7.4 Samples containing collagen of 0.2 wt %, 0.3 wt %, 0.4 wt % and 0.5 wt % concentrations in αMEM were obtained, which are usually used in cell culture experiments. All reagents were kept on ice during sample preparation and the polymerization was initiated by bringing the sample to desired temperature using a temperature controlling system. A minimal amount of silicon oil was added to the free surface of the sample to prevent evaporation.

### III-D. Mechanical characterization of hydrogels: Theory and method

In this research, we report a case study of the mechanical characterization especially shear modulus measurement of the agarose and collagen gels of various concentrations that has been carried out using the constructed magnetic bead rheometry device. The microscope-based magnetic bead rheometer, which was equipped to an internal soft-iron based core tip, can apply magnetic field and hence, mechanical shear force on the magnetic particle, and the corresponding displacement of the magnetic bead can be measured. The determination of these two parameters allows us to calculate the linear elasticity parameter namely elastic shear modulus. Classical linear elasticity theory was then employed to derive the force-displacement relation necessary to process the measurement data. The obtained shear modulus values are compared with the results of the previous studies reported in the literature.

Hydrogels, despite the quasi-liquid-like behavior, are functionally solids and are thus assumed to be perfectly elastic for the present study. The mechanical behavior of hydrogel is thus best understood by theory of elasticity. This theory is based on the time-independent recovery of the chain orientation and structure. Elasticity indeed is introduced as the physical property of a material by virtue of which it returns to its original shape after the force under which it deforms is removed. The applied force is usually referred to as stress, which is the force acting per unit cross-sectional area of the material, while the relative deformation is called as strain. The elastic regime is characterized by a linear relationship between stress and strain. The ratio of stress to strain is constant for a given material and is the defining mechanical property of material. When the applied force is parallel to the area supporting it, the stresses and strains are shear, which the proportionality constant obtained for the ratio of the shear stress to shear strain is referred to as shear modulus. Considering that the biopolymer-based hydrogels are comparatively isotropic and homogeneous and also regarding the elasticity theory which assumes that when a stress is applied to the hydrogel, the strain response is instantaneous, the hydrogel shear modulus is measured using the constructed microscope-based magnetic bead rheometry set-up based on stress–strain relation.

**Fig 6:**
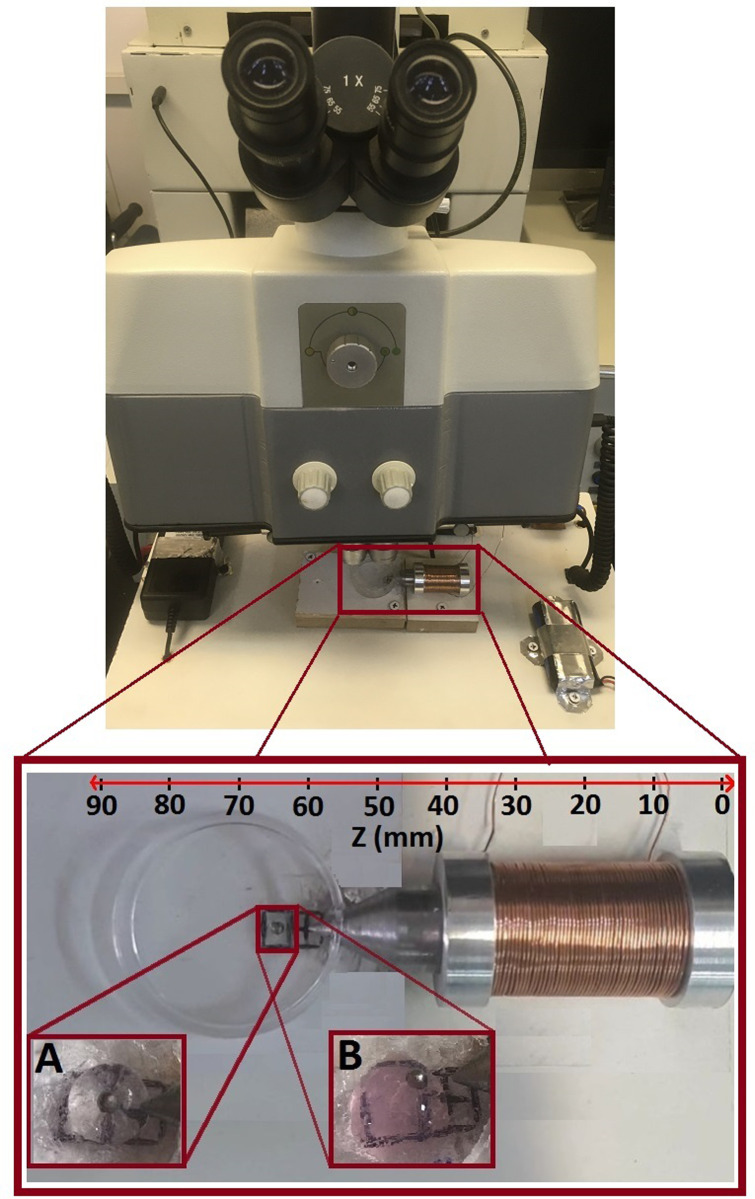
The prepared samples of bead and hydrogels of agarose and collagen, as inset pictures of A and B respectively, within water in the vicinity of the magnetic core tip of AMBR set-up.

Considering the force calibration of the setup, the gel samples of 3–4 mm diameters were placed in distances up to 3 mm to the electromagnet core tip in the cylindrical polystyrene holder. The magnetic particle of 1000 μm was then placed on the top of the prepared semispherical hydrogel sample with which have a perfect surface adhesion. After determining the distances between magnetic particle and electromagnet, core tip was accurately aligned with the magnetic bead to be completely exposed to the generated magnetic field before the set-up start to operate. Fig 6 shows the bead and hydrogel samples of Agarose (inset picture A) and Collagen (inset picture B) in water in the vicinity of electromagnet core tip that was prepared for the measurement of gel shear modulus.

The magnetic field was generated in the vicinity of the magnetic particle by applying the different current values ranging from 2500 mA to 4000 mA to the electromagnet, that were experimentally measured by using the provided multimeter. Since the magnetic bead was placed in the distances of 1–3 mm to the magnetic core tip, the magnetic field was thus assumed to be uniform in such low distances. Therefore, a constant magnetic force up to 3 μN for each specific current value will act on the magnetic particle due to the applied magnetic field leading to the bead displacement toward the magnetic core tip. The complete adhesion of the bead and the gel surface cause to the magnetic force, acting on the bead of 1000 μm, be applied on the gel surface in the form of mechanical shear stress. Indeed, the most important advantage of this method as compared with the other techniques is that the hydrogel surface is directly exposed to the real mechanical shear forces due to the presence of the placed magnetic particle on its surface, allowing us to calculate the gel shear modulus using the stress-strain relations which is mentioned in Eq 2:

$$\tau =\frac{\text{F}}{\text{A}},\;\gamma =\frac{\text{X}}{\text{h}},\;\text{G}=\frac{\tau }{\gamma }$$

$$\text{G}=\frac{\text{F}.\text{h}}{\text{X}.\text{A}}$$ (2)

In which τ, γ, F, h, X, A and G are respectively shear stress, shear strain, the applied magnetic force, gel thickness, bead displacement, cross sectional area of shear stress, and hydrogel shear modulus.

The magnetic force (F) which is applied by the calibrated electromagnet to the placed bead on the gel surface can be calculated regarding the force calibration results by the determination of the magnetic bead distance to the core tip and the experimentally measurement of the electric current using the multimeter. Since the magnetic force, acting on the magnetic bead, is immediately transmitted in the form of mechanical shear force to the hydrogel surface; the approximate adhesion area of the bead of 1000 μm and hydrogel is assumed to be considered as the cross sectional area (A) of this mechanical-magnetic force. Eventually, considering the initial place of the particle, the bead displacement (X) toward the electromagnet core tip can be accurately measured by a camera-based-microscope using the image magnification technique even for the small movements. By the determination of the effective parameters in Eq 2, the elastic shear modulus of the hydrogels can be then calculated.

In addition, a series of experimental tests were performed in two distinct steps in order to investigate the accuracy and the repeatability of the mechanical characterization results of this research. First, the agarose and collagen gels of different percent concentrations were prepared in two different times and the mechanical characterization tests were separately repeated to minimize the effect of the probably occurred differences in the hydrogel preparation. In the second step, the characterization tests were at least twice repeated for each gel concentration in order to decrease the errors that probably occurred in the experiments. In both cases, it was observed that the same results, with lower than 5% difference, were obtained.

The mechanical characterization tests were eventually carried out for the agarose and collagen gels of different concentrations in different electric currents ranging from 3000 mA to 4000 mA and the graphs of shear stress versus shear strain were then plotted for each hydrogel percent concentration. The average shear modulus of the hydrogels was also computed using linear fitting technique. In addition, the obtained shear modulus of the agarose and collagen hydrogels of different percent concentrations were compared for better analysis of the gel concentration effect on its mechanical properties.

## IV. Results and discussions

We have presented an experimental study of the effect of agarose and collagen gel concentration on hydrogel mechanical properties. We have focused on the hydrogel strength and linear elastic behavior as function of agarose and collagen percent concentration. Although the effect of agarose and collagen concentration has been recently studied [31–38], we have in this fine-tuned the concentration reach mechanical properties of specific use for neural and bone tissue engineering. We have accomplished this via in-home-built Amirkabir Magnetic Bead Rheometry set-up.

At first, the agarose gel samples of 0.2 wt %, 0.3 wt %, 0.4 wt %, 0.5 wt %, and 0.6 wt % percent concentrations were experimentally tested to calculate the gel elastic shear modulus parameter, which describes the material’s elastic response to shear stress.

**Fig 7:**
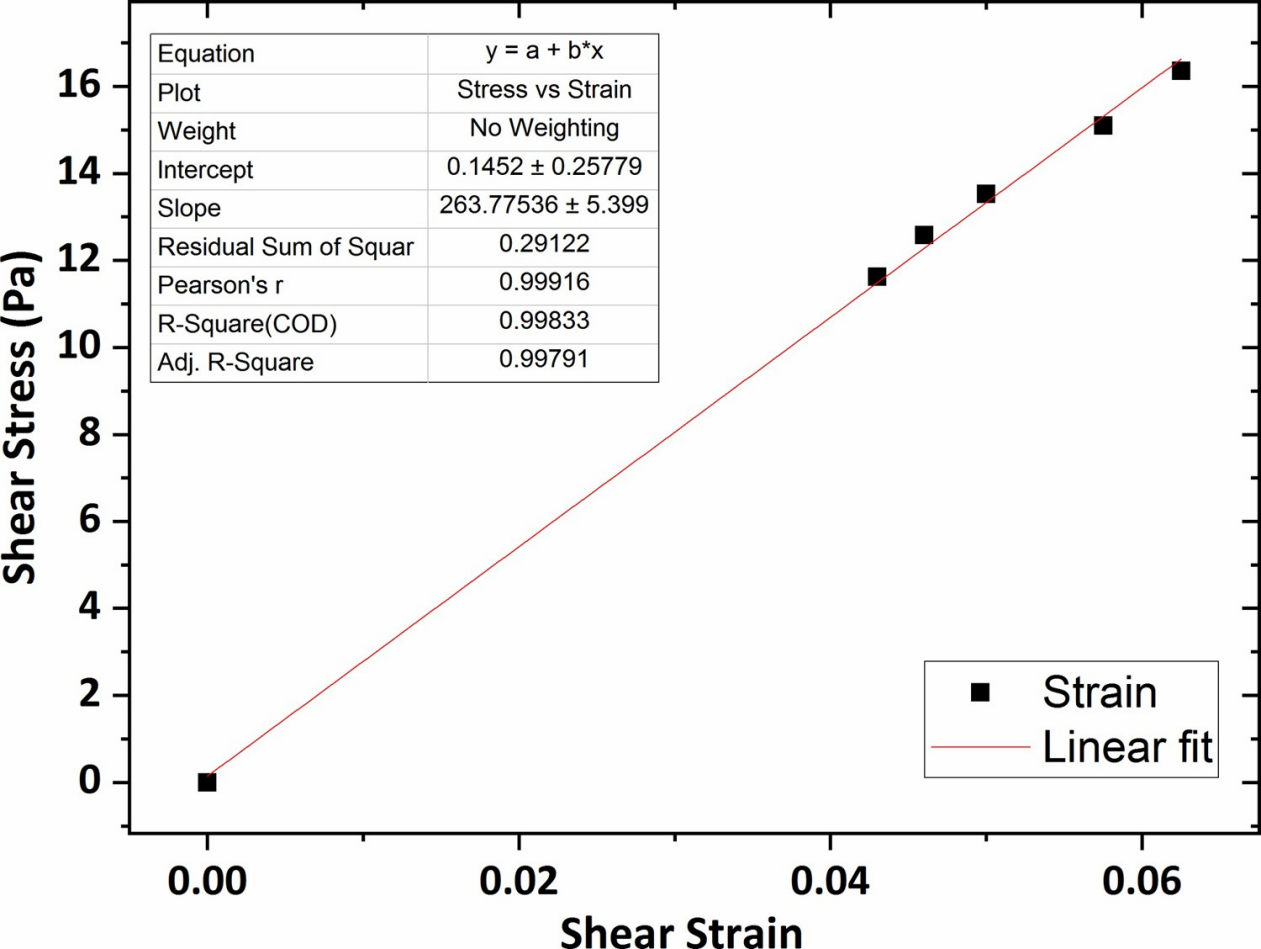
The graph of shear stress versus shear strain for the agarose gel of 0.2 wt % percent concentration.

**Fig 8:**
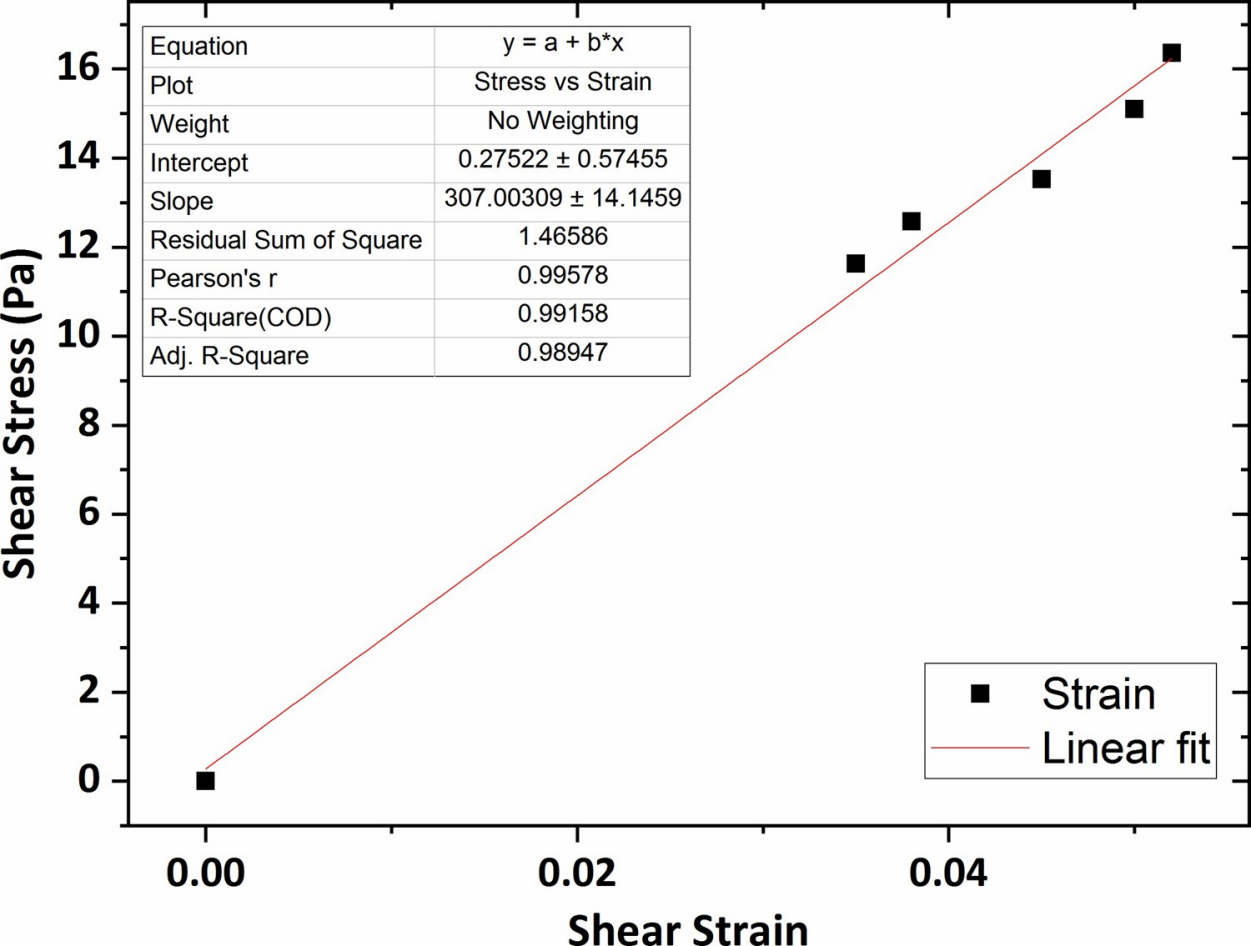
The graph of shear stress versus shear strain for the agarose gel of 0.3 wt % percent concentration.

Fig 7 shows the graph of the shear stress versus shear strain for the agarose hydrogel of 0.2 wt % percent concentration in which the average shear modulus (G) was obtained 264 Pa using linear fitting technique. The shear modulus value for the agarose gel of 0.3 wt % concentration was obtained 307 Pa, shown in Fig 8.

**Fig 9:**
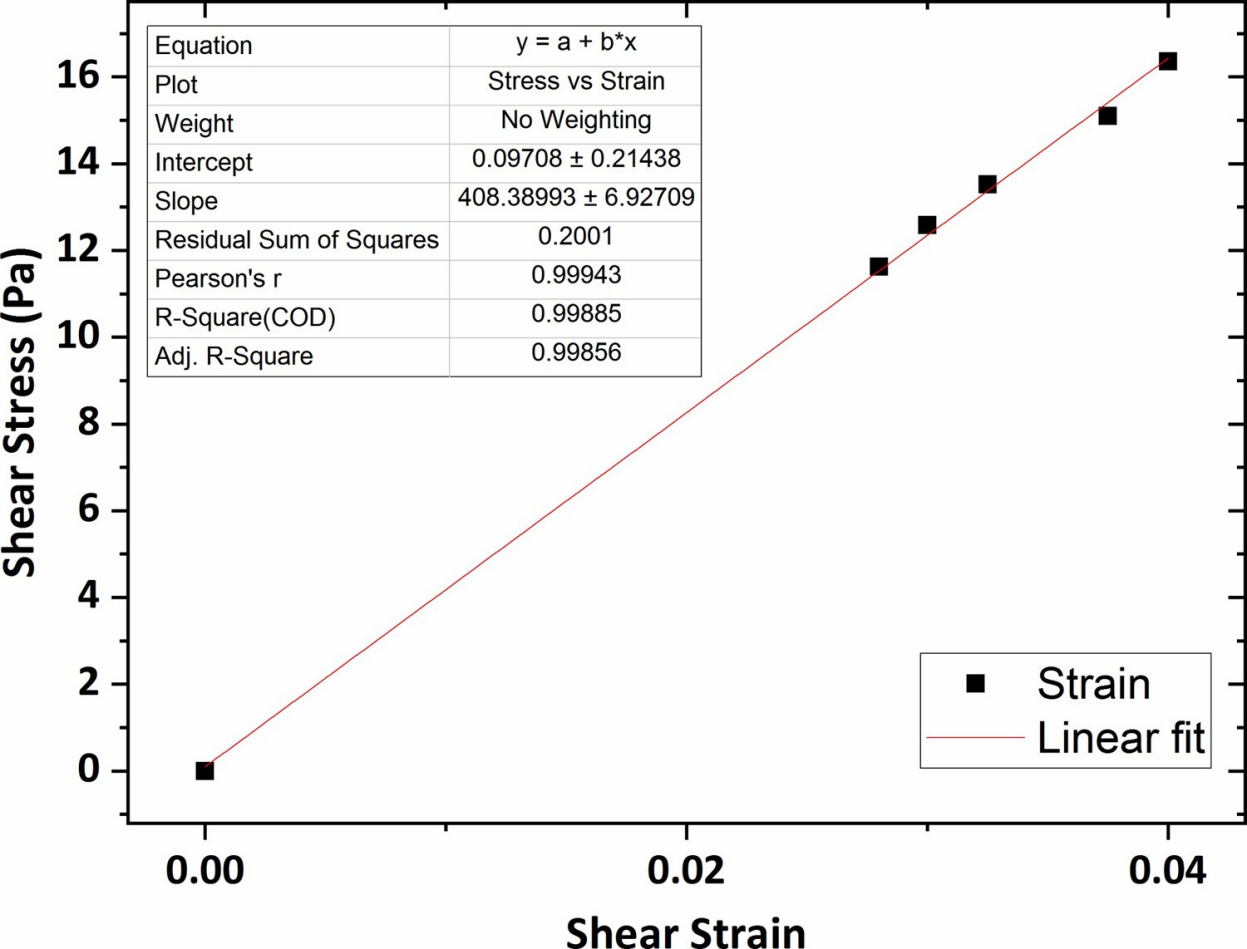
The graph of shear stress versus shear strain for the agarose gel of 0.4 wt % percent concentration.

**Fig 10:**
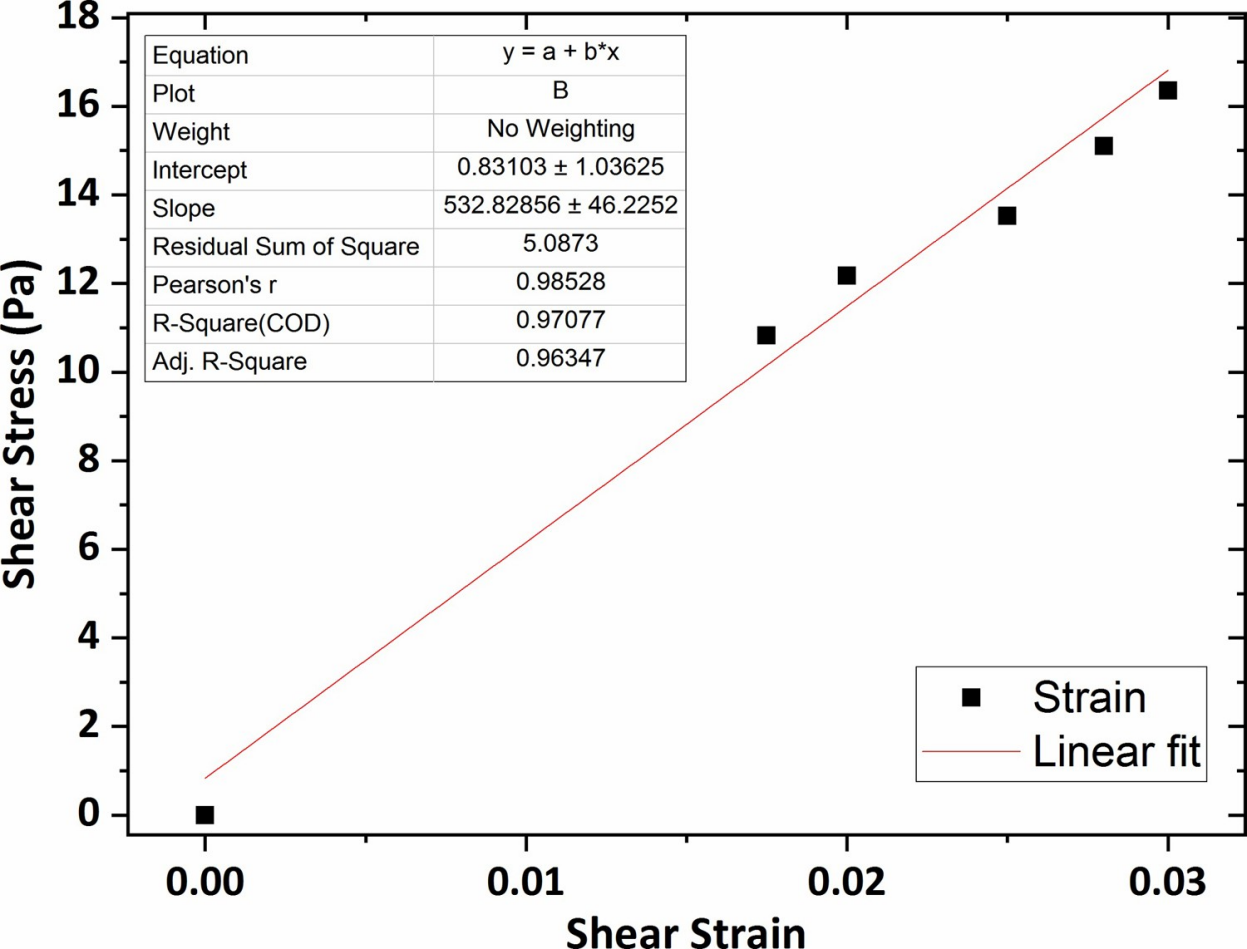
The graph of shear stress versus shear strain for the agarose gel of 0.5 wt % percent concentration.

Similarly, the graphs of shear stress versus shear strain for the agarose gel of 0.4 wt % and 0.5 wt % concentrations, plotted in Figs 9 and 10, show that the average shear modulus was respectively obtained 408 Pa and 532 Pa.

**Fig 11:**
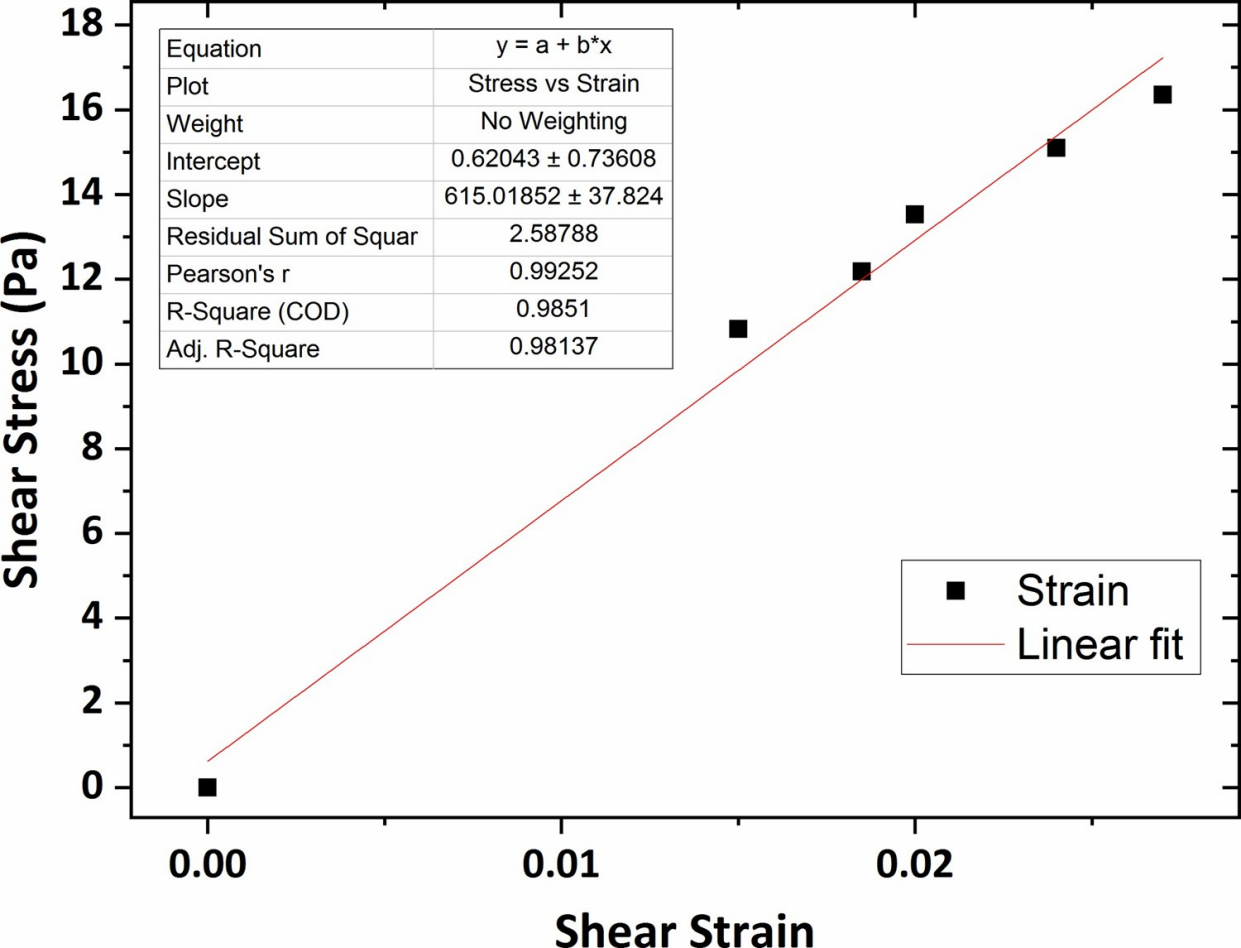
The graph of shear stress versus shear strain for the agarose gel of 0.6 wt % percent concentration.

Eventually, the shear modulus value for the agarose gel of 0.6 wt % concentration, the highest concentration of agar gel in this research, was obtained 615 Pa that was shown in Fig 11.

**Fig 12:**
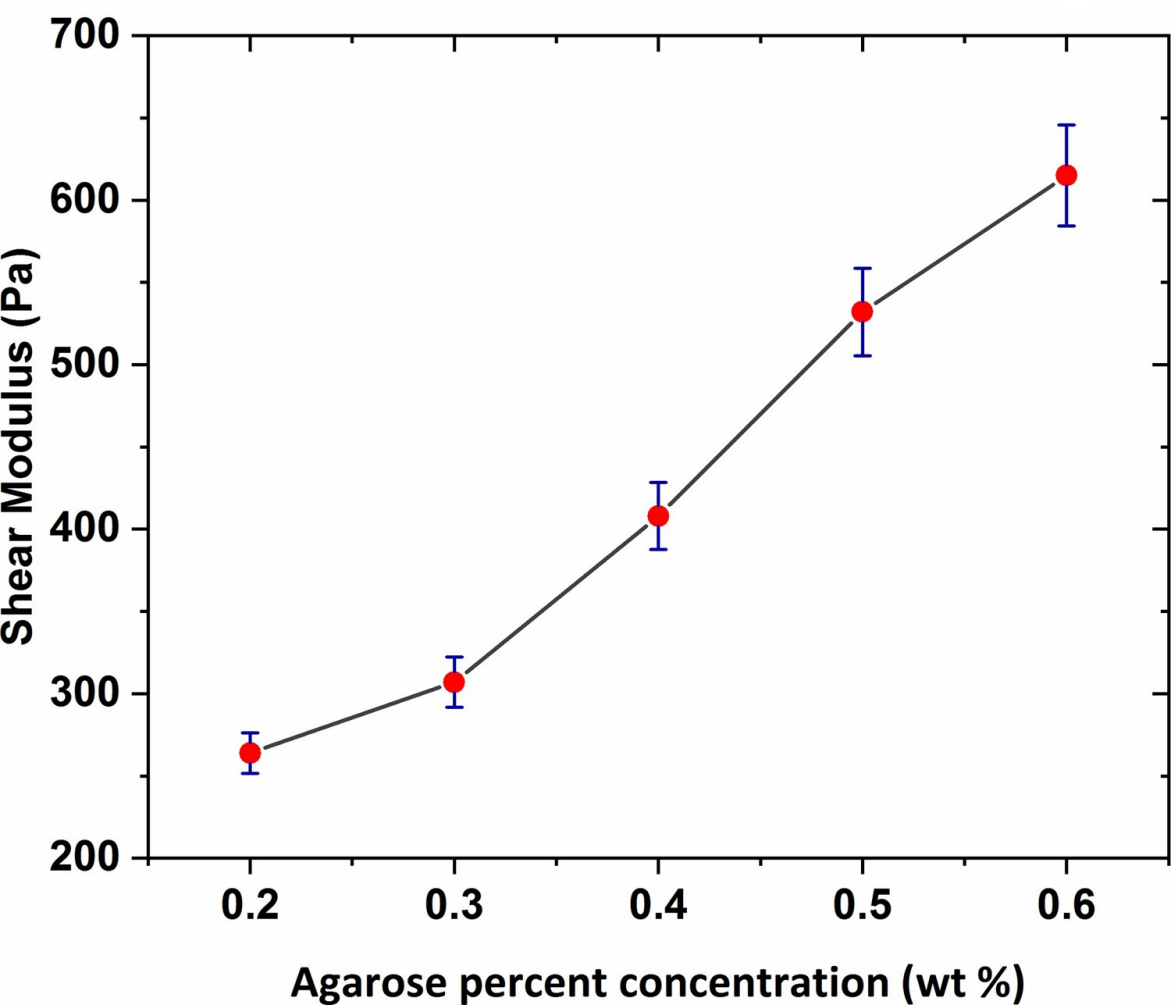
The graph of elastic shear modulus of agarose hydrogel versus agar percent concentration with error bars based on a 95% confidence interval from the linear fitting procedure.

The results of the shear modulus, measured with varying agar gel concentration, are shown in Fig 12 with error bars based on a 95% confidence interval from the linear fitting procedure. As observed in the figure, the shear modulus value increased slightly for concentrations ranging between 0.2 wt % to 0.3 wt % and then for the rest of the range, a significant increase was seen by increasing the gel concentration reaching over 0.6 KPa at concentration of 0.6 wt %.

As observed, the average shear modulus of agarose gel samples was obtained in the range 250–650 Pa. As expected, the shear modulus was higher as the agarose concentration increased. In previous studies, that were particularly performed for the measurement of the agarose gel vs. shear modulus, a shear modulus at 800–1500 Pa in the range 0.6–1 wt % was reported for agarose. The shear modulus values of the agarose gel samples of 0.6 wt % and 0.65 wt % percent concentrations were also obtained in the range 700–1000 Pa as mentioned in Refs [50] and [51]. Although the direct comparison is not possible, considering the gel concentration effect on the mechanical characteristics of hydrogels, it can be expected the shear modulus of the agarose gel of 0.2–0.6 wt % concentration to be obtained in the range 300–600 Pa. Therefore, the obtained shear modulus in this research with considering the gel concentration effect on hydrogel shear modulus and the probable differences in experimental methods, are in an approximately good accordance with the previous results. Mechanical properties of human brain tissue have also been studied *in-vivo* and *in-vitro* using rheology-based techniques by some earlier studies. For instance, Fallenstein et al [32] have studied the shear modulus of *in-vitro* samples of human brain reporting elastic shear modulus value in the range 600–1100 Pa in accordance with our values for agarose gel of concentration 0.5 wt % and 0.6 wt %. As a result, these specific agarose gel samples have mechanical properties comparable to those of human brain tissue. From the comparison of elastic shear modulus, we conclude that the agarose gel material of concentration 0.6 wt % is suitable to be used as brain tissue mimicking material.

The results of the mechanical characterization especially elastic shear modulus measurement of different collagen concentrations of 0.2 wt %, 0.3 wt %, 0.4 wt %, and 0.5 wt % are also reported herein. The collagen-based hydrogels here characterized have been previously used to culture fibroblasts, osteoblasts, and processes such as cell migration have been proved to evaluate correctly using these gels and cell viability was confirmed in all cases.

**Fig 13:**
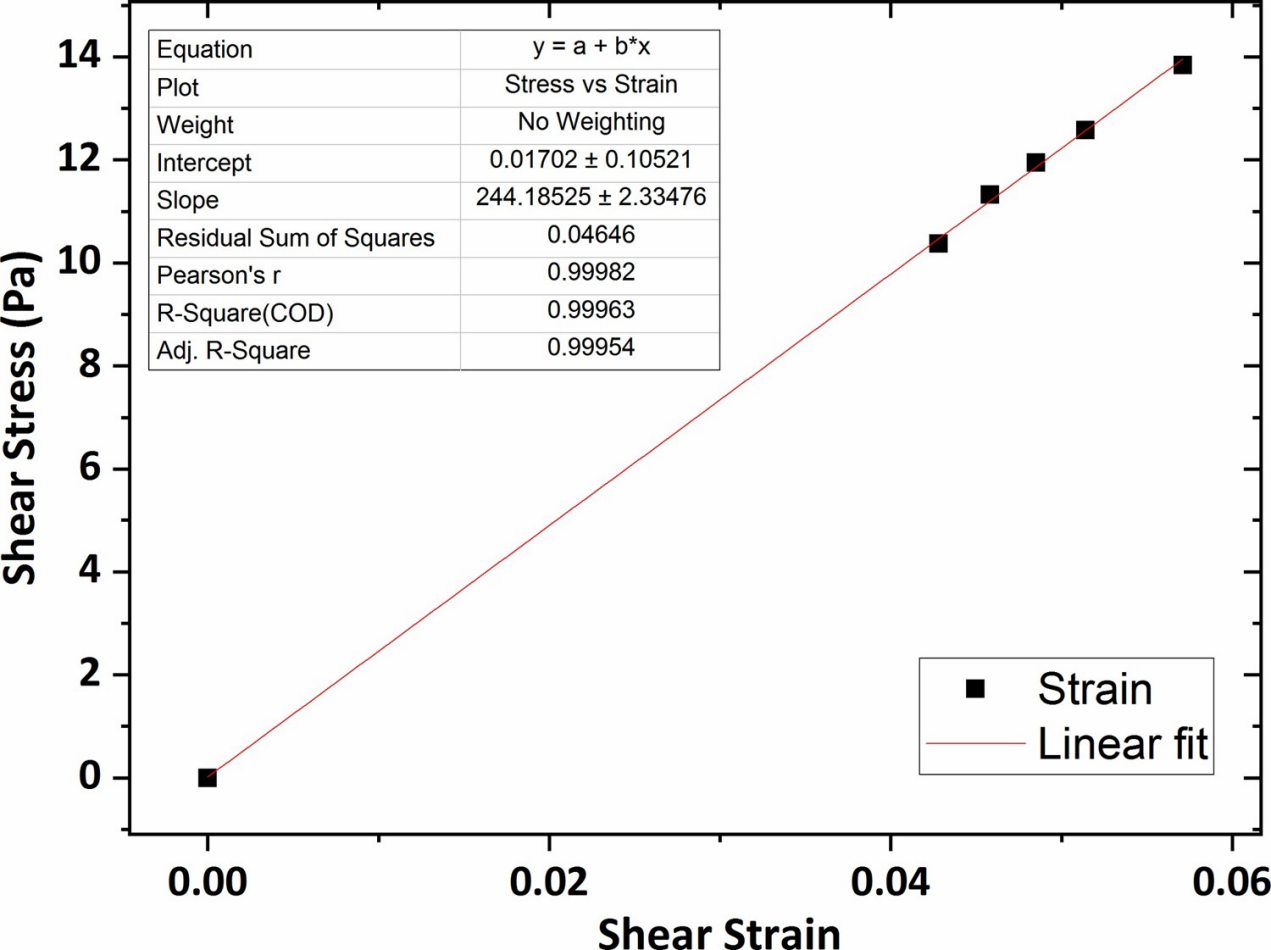
The graph of shear stress versus shear strain for the collagen gel of 0.2 wt % percent concentration.

**Fig 14:**
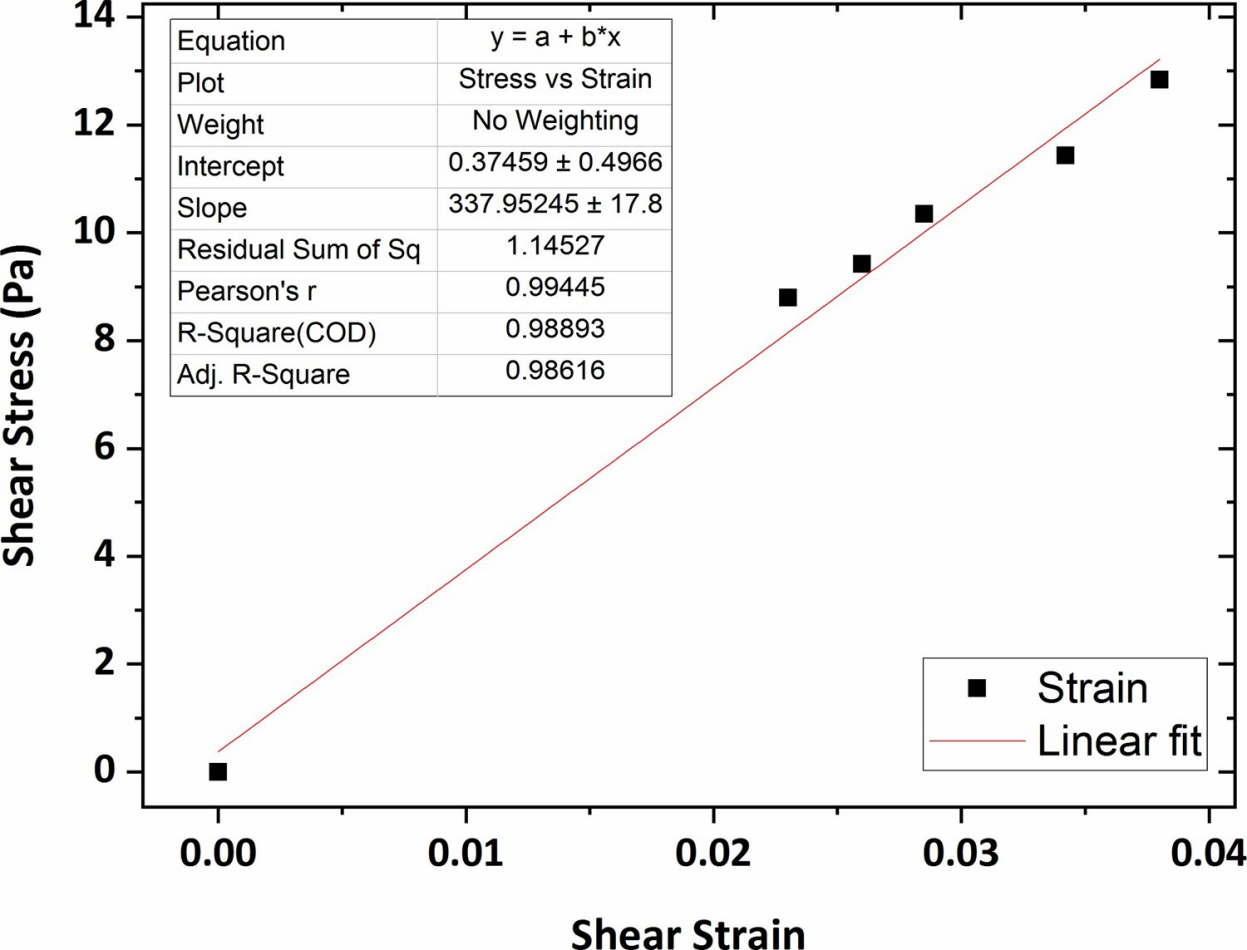
The graph of shear stress versus shear strain for the collagen gel of 0.3 wt % percent concentration.

**Fig 15:**
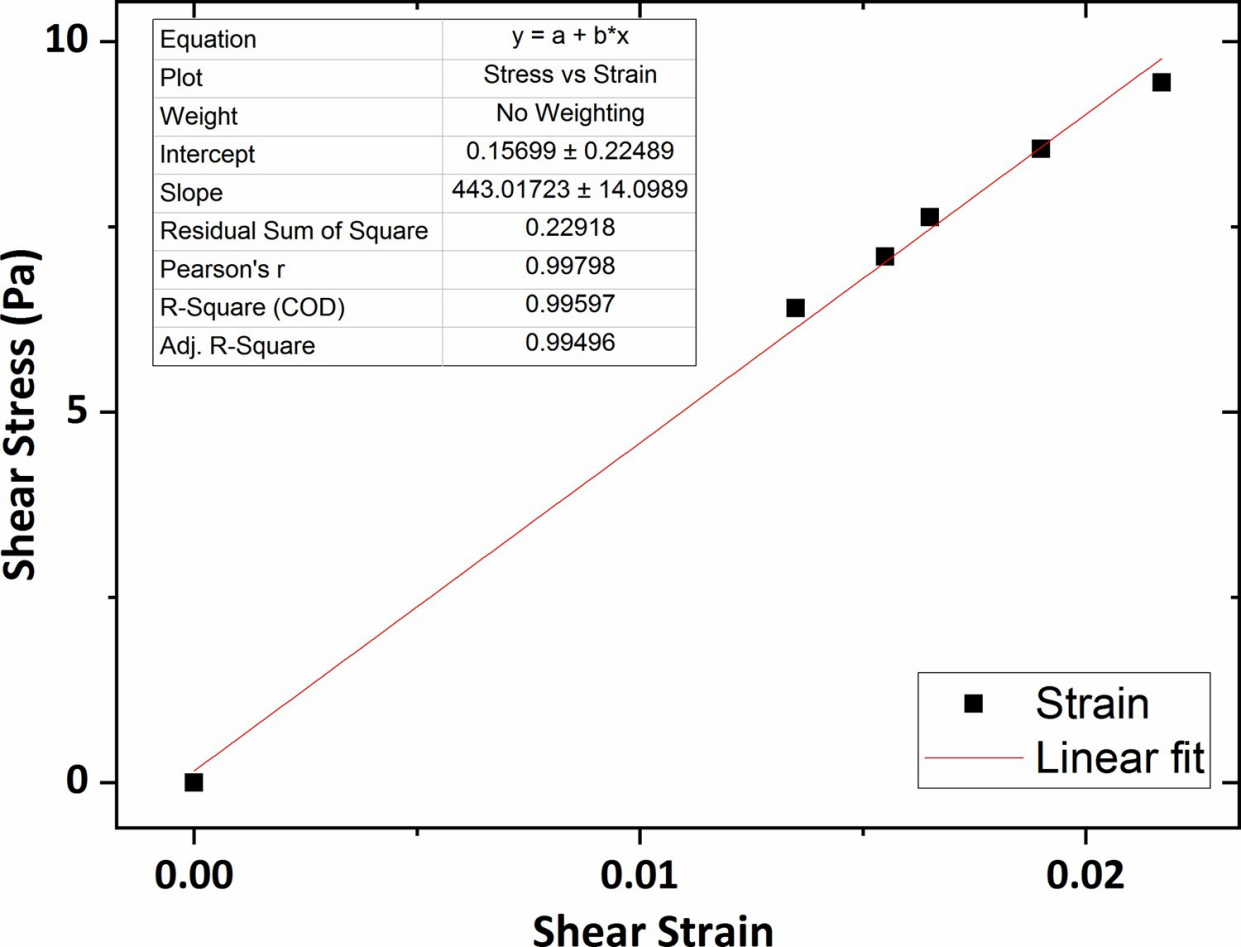
The graph of shear stress versus shear strain for the collagen gel of 0.4 wt % percent concentration.

Fig 13 shows the graph of the shear stress versus shear strain for the collagen hydrogel of 0.2 wt % percent concentration in which the average shear modulus was obtained at 244 Pa using linear fitting technique. In addition, according to Figs 14 and 15, the average shear modulus of the collagen gel of 0.3 wt % and 0.4 wt % percent concentration was obtained respectively 338 Pa and 443 Pa.

**Fig 16:**
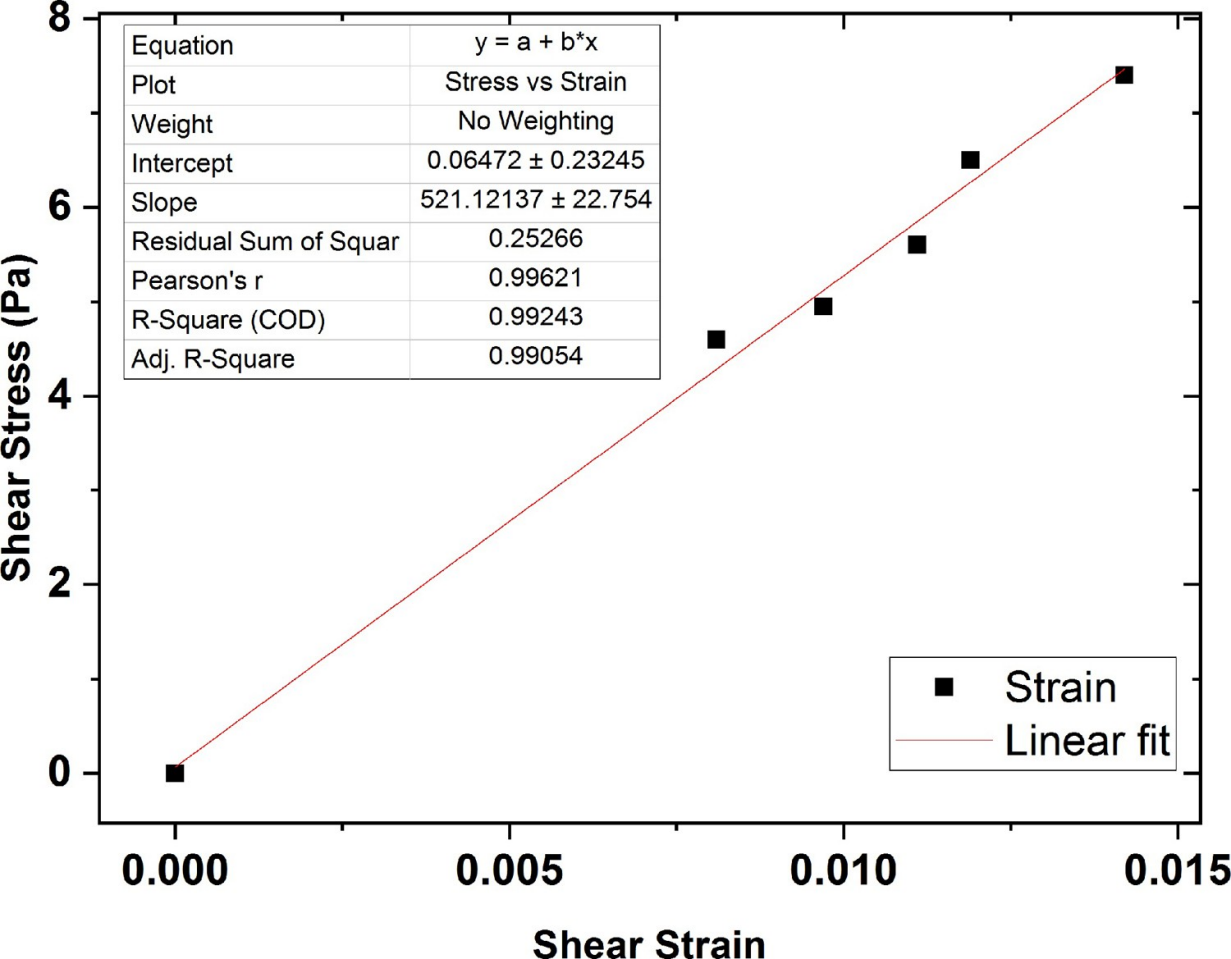
The graph of shear stress versus shear strain for the collagen gel of 0.5 wt % percent concentration.

The average shear modulus value for the collagen gel sample of 0.5 wt % concentration was also obtained 520 Pa, shown in Fig 16.

**Fig 17:**
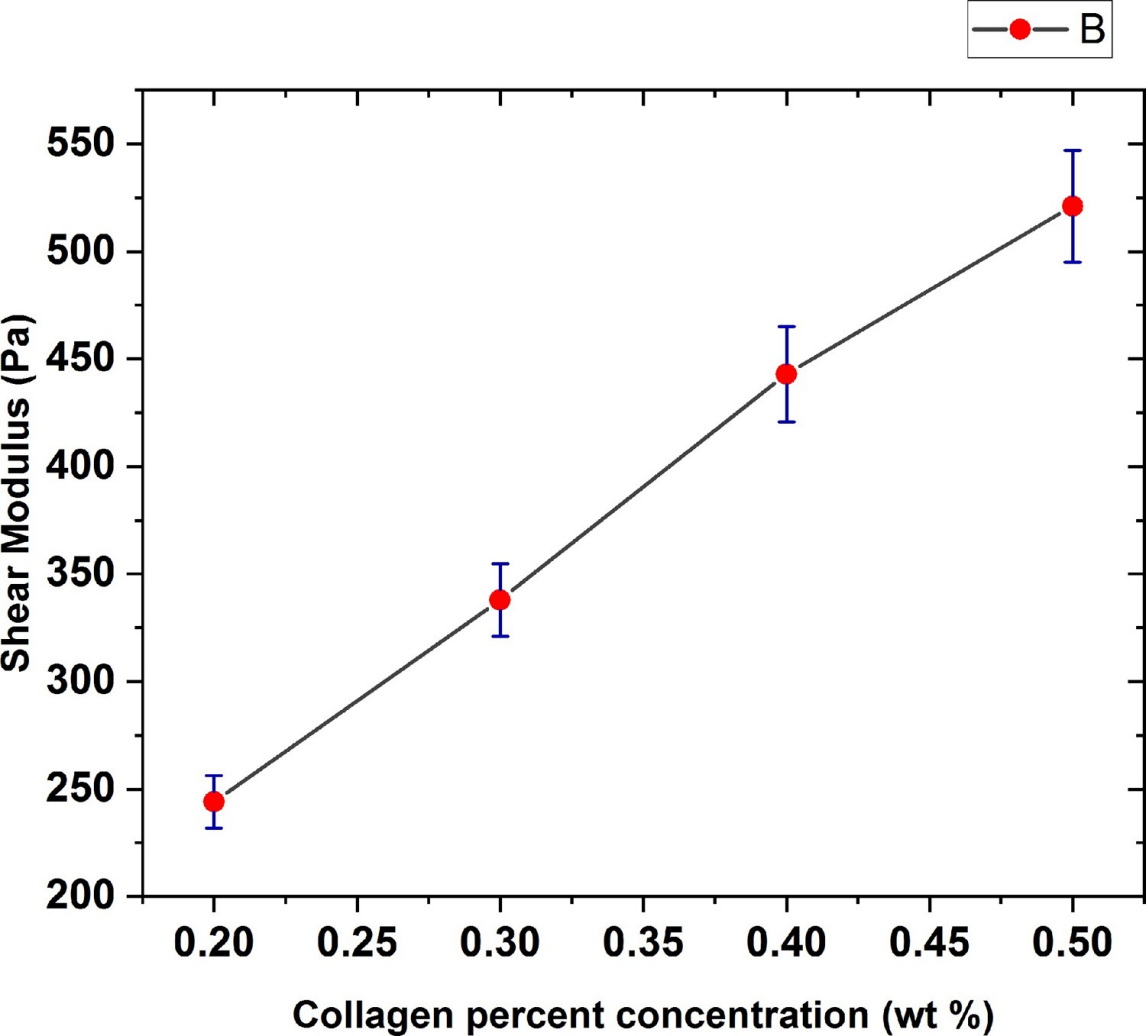
The graph of elastic shear modulus of collagen hydrogels versus gel percent concentration with error bars based on a 95% confidence interval from the fitting procedure.

For better analysis of the collagen gel concentration effect on the hydrogel mechanical characteristics, the shear stress variations versus the shear strain were plotted in Fig 17 with error bars based on a 95% confidence interval from the fitting procedure. As seen in the figure, the collagen shear modulus increases in an approximately linear fashion by increasing in hydrogel percent concentration, indicating gels of varying collagen concentration show different mechanical and structural properties.

As expected, the shear modulus increased with collagen concentration, ranging from 200–550 Pa for 2 to 5 mg/ml. The previous studies [38], in which rheometry was performed to evaluate the initial stiffness of the gels of varying collagen, particularly in "no cell" condition, reported that the shear modulus values of the collagen hydrogel of 0.2–0.4 wt % percent concentrations were obtained in the range 200–500 Pa which agrees with our results. It seems that the small differences that are observed between the previous and the present results are due to using the different methods for the collagen hydrogel shear modulus measurement.

We found that the shear modulus of the polymerized gels present a linear dependence on the collagen concentration within the tested range. In other words, the shear modulus value was higher as the collagen concentration increased. The main effect of increasing the collagen concentration was the increase of the hydrogel stiffness and strength. When collagen concentration is higher, more fibers are formed and they are more densely packed resulting in a stiffer material. Indeed, results demonstrate a key point which states collagen with higher concentration forms firmer hydrogels indicating that higher shear stresses are required for the same shear deformation. This fact has major implications for extracellular matrix (ECM) and hence, biological processes since matrix stiffness can impact a wide variety of cellular pathways including propagation, differentiation, migration, self-renewal, morphology, and gene expression. In addition to this, an excessive collagen production may cause an excessive rigid tissues which probably alter the regular cell behavior. On the other hand, the structural integrity of a hydrogel construct may be of importance, particularly in load bearing scenarios. It is thus so important to fabricate bioengineered tissues or substitutes showing the desired mechanical properties closest to those of natural tissue, which is considered as the most important aim of the related researches to tissue engineering discipline.

The possibility of the fabrication of agarose and collagen hydrogels with the desired mechanical characteristics makes them suitable for a large number of biomedical applications. Agarose hydrogels due to their non-immunological feature and tunable mechanical properties, can be used to mimic extracellular matrix and emulate tissues such as human brain tissue. Collagen hydrogels (type I) are also widely utilized in cell culture systems and three dimensional scaffolds in tissue engineering due to their biocompatibility with cells and their capacity to mimic biological tissues. The mechanical properties specifically mechanical stiffness of collagen-based substrates plays a crucial role in regulation of cell behavior. The determination of the mechanical properties in particular shear modulus of hydrogels will eventually provide a novel insight into the mechanics of cell substrate, enabling us to better modulate cell biological responses.

## V. Conclusion

Herein, the microscope-based Amirkabir Magnetic Bead Rheometry (AMBR) set-up was successfully constructed and utilized to measure shear modulus of agarose and collagen hydrogels of different concentrations in a non-destructive fashion. The set-up of this work enable us to characterize hydrogel mechanical properties in terms of shear modulus without such limitation in prepared gel sample dimensions in condition closest to cell culture media. The specific design of an optimized electromagnet equipped to a conical-shaped core tip, cause to generation of high intensity focused magnetic fields in the vicinity of the magnetic bead leading to the application of magnetic forces with tens of μN magnitude to actuate magnetic beads in contact with the gel surface in order to actuate the gel itself. The average shear modulus of the agarose gel of 0.2 wt %, 0.3 wt %, 0.4 wt %, 0.5 wt %, and 0.6 wt % percent concentrations was respectively obtained 264 Pa, 307 Pa, 408 Pa, 532 Pa, and 615 Pa which is in a good accordance with the reported results of the previous studies. Also, it was observed the agarose gel shear modulus increased by increasing gel percent concentration which reaches 0.6 KPa for the agarose gel of 0.6 wt % concentration. From the comparison of the shear modulus, it can be concluded that the agarose gel samples of concentration 0.5 wt % and 0.6 wt % are suitable to be used as brain mimicking biomaterial. In addition, the shear modulus values of the collagen gel of 0.2 wt %, 0.3 wt %, 0.4 wt %, and 0.5 wt % percent concentrations were obtained respectively at 244 Pa, 338 Pa, 443 Pa, 520 Pa; in accordance with a collagen shear modulus increases as function of gel concentration. The determination of the shear modulus of collagen gel samples will eventually provide a novel insight into the mechanics of collagen-based cell substrate, enabling us to better regulate cell biological behavior. Using in-home-built AMBR set-up for experimental measurement of cell mechanical properties in terms of shear modulus and also investigation of the cellular mechanical response to the applied mechanical stimulations are the topics of our future work.
